# Therapeutic efficacy of albuvirtide-based antiretroviral therapy in people living with HIV who have low-level viremia and non-AIDS-defining malignancies: two case reports

**DOI:** 10.1186/s12977-025-00662-5

**Published:** 2025-04-18

**Authors:** Chuantiao Zhang, Tingting Xie, Yuantao Liu, Yang Cao

**Affiliations:** 1 Department of Infectious Diseases, Shishi Municipal Hospital, No.2156 Shishi Road, Shishi, Quanzhou, 362700 Fujian China; 2 Medical Affairs Department, Frontier Biotechnologies Inc, Nanjing, Jiangsu China

**Keywords:** HIV, Low-level viremia, Albuvirtide, Non-AIDS-defining cancers, Antiretroviral therapy, Case report

## Abstract

**Background:**

People living with HIV (PLWH) who experience low-level viremia (LLV) face unique challenges in disease management, particularly when diagnosed with concurrent malignancies. Albuvirtide (ABT), a long-acting HIV fusion inhibitor approved in China, has shown promise in clinical trials for treatment-experienced individuals. However, its efficacy in managing LLV in the context of concurrent malignancies remains under-explored.

**Case presentation:**

We report two cases of PLWH with LLV who developed non-AIDS-defining cancers(NADCs). The first individual developed lung squamous cell carcinoma, and the second was diagnosed with breast cancer. Both patients received ABT as part of their optimized antiretroviral therapy (ART) regimen during their cancer treatment course. After treatment optimization, both cases achieved viral suppression (HIV-1 RNA < 50 copies/mL) with improvements in CD4 + T cell counts. Both patients received appropriate cancer treatments according to clinical practice guidelines. The patient diagnosed with lung cancer required an adjustment to his PD-1 inhibitor monotherapy due to intolerance to chemotherapy, whereas the breast cancer patient successfully completed her planned multimodal treatment regimen.

**Conclusions:**

These cases suggest potential benefits of ABT-containing ART regimens in PLWH who have LLV and concurrent NADCs. While two cases cannot establish definitive conclusions, they highlight the need for larger studies investigating the role of ABT in this complex clinical scenario.

## Background

The clinical management of human immunodeficiency virus (HIV) has evolved significantly with the advent of combination antiretroviral therapy (ART), transforming HIV from a fatal illness to a chronic, manageable condition. However, a subset of people living with HIV (PLWH) continue to experience low-level viremia (LLV), defined as persistent plasma HIV-1 RNA levels between 50 and 1000 copies/mL [1]. Current clinical practice guidelines emphasize the importance of regular viral load monitoring, thorough medication adherence assessment, and careful evaluation of potential drug resistance patterns in managing LLV. When clinically indicated, healthcare providers conduct genotypic resistance testing and optimize ART regimens based on individual treatment history and resistance profiles [2].

The etiology of LLV is multifactorial, encompassing challenges with medication adherence, periodic release of virus from latently infected cells, and possible ongoing viral replication in sanctuary sites [3–7]. Recent evidence suggests that LLV contributes significantly to chronic immune activation and inflammation, which may increase the risk of non-AIDS-related comorbidities, including malignancies [8]. The persistent immune activation associated with LLV can potentially compromise the body’s natural tumor surveillance mechanisms and affect overall clinical outcomes.

The management of HIV becomes particularly complex when PLWH with LLV are diagnosed with concurrent malignancies. Non-AIDS-defining cancers (NADCs) occur at notably higher rates in PLWH compared to the general population, with current epidemiological data showing standardized incidence rate ratios (SIRs) ranging from 1.5 to 3.0 [9]. The comprehensive clinical management of NADCs in PLWH requires careful attention to potential drug interactions between antiretroviral medications and cancer therapeutics, alongside continuous monitoring of immune function throughout cancer treatment [10]. The coordination between HIV specialists and oncologists becomes crucial in optimizing both HIV suppression and cancer treatment outcomes [11].

Albuvirtide (ABT), a long-acting HIV fusion inhibitor approved in China, represents an important therapeutic option for treatment-experienced PLWH. The drug is specifically indicated for use in combination with other antiretroviral agents in individuals who have documented resistance to multiple antiretroviral drug classes or face challenges with daily medication adherence [12, 13]. The mechanism of action involves binding to the gp41 subunit of the HIV envelope glycoprotein, specifically targeting the HR1 region. This interaction prevents the formation of the six-helix bundle structure necessary for viral fusion with host cells [14, 15].

The pharmacological profile of ABT offers several potential advantages in clinical practice. Its extended half-life of approximately 11–12 days enables once-weekly dosing, which may significantly improve medication adherence among PLWH [16]. Detailed pharmacokinetic studies have demonstrated that ABT achieves steady-state plasma concentrations after 2–3 doses, maintaining therapeutic levels above the protein-binding adjusted IC50 for wild-type HIV-1 throughout the weekly dosing interval [17]. This sustained therapeutic concentration may be particularly beneficial in the context of LLV, where consistent viral suppression is crucial.

While clinical trials have established ABT’s efficacy in treatment-experienced PLWH, its specific role in managing LLV concurrent with cancer treatment remains an important area for investigation. The intersection of HIV management and cancer treatment presents unique challenges, including potential drug interactions, immune system modulation, and the need to maintain optimal viral suppression during cancer therapy [18]. Understanding how ABT-containing regimens perform in this complex clinical scenario could provide valuable insights for optimizing care in this vulnerable patient population.

In this context, we present two cases of PLWH with LLV who developed different types of NADCs and received ABT as part of their optimized ART regimen alongside cancer treatment. These cases provide preliminary observations regarding albuvirtide-based antiretroviral therapy use in this clinical setting, suggesting areas for further investigation through larger, systematic studies in this patient population.

### Case presentation

#### Case 1: HIV-infected patient with lung cancer

A 57-year-old man was diagnosed with HIV infection in April 2021, with an initial HIV-1 RNA of 1.04 × 10⁵ copies/mL and CD4 + T cell count of 64.66 cells/µL. In May 2021, he initiated antiretroviral therapy with tenofovir disoproxil fumarate, lamivudine, and efavirenz. By February 2023, laboratory tests showed HIV-1 RNA had decreased to 113 copies/mL and CD4 + T cell count had increased to 283 cells/µL, although low-level viremia persisted. Genotypic resistance testing at this time revealed the K103N mutation in the reverse transcriptase gene, indicating resistance to non-nucleoside reverse transcriptase inhibitors (NNRTIs) including efavirenz. Medication adherence assessment using the AIDS Clinical Trials Group (ACTG) adherence questionnaire showed an 82% self-reported adherence rate, with occasional missed doses attributed to work schedule conflicts.

The patient’s clinical course became more complex in August 2023, when routine chest imaging revealed a left lung mass. Subsequent diagnostic evaluation, including biopsy and immunohistochemistry, confirmed lung squamous cell carcinoma with TPS 40% (tumor proportion score) PD-L1 expression. Concurrent disease assessment via contrast-enhanced chest CT demonstrated significant disease burden, with a left hilar tumor measuring 89 mm × 66 mm, multiple pulmonary nodules, and evidence of hepatic metastases, corresponding to stage IVB (cT4N2M1c) disease. In November 2023, he initiated combination therapy with a PD-1 inhibitor and chemotherapy. The initial regimen consisted of carboplatin (AUC 5, day 1) and paclitaxel (175 mg/m², day 1) administered in 21-day cycles, combined with pembrolizumab (200 mg, day 1). During chemotherapy, the patient developed Grade 3 myelosuppression (neutrophil count 0.8 × 10^9^/L) and Grade 2 peripheral neuropathy according to CTCAE v5.0 criteria. Despite dose reduction attempts, these adverse events persisted.

During this same period, the patient experienced a concerning deterioration in HIV suppression, with HIV-1 RNA increasing to 233 copies/mL and CD4 + T cell count declining precipitously to 22.82 cells/µL. In response to this clinical deterioration, the patient’s antiretroviral regimen was modified to incorporate albuvirtide (320 mg per dose, administered on days 1, 2, 3, and 8, followed by weekly maintenance doses) in combination with efavirenz, lamivudine, and tenofovir alafenamide.

Due to chemotherapy intolerance, cancer treatment was subsequently adjusted to PD-1 inhibitor monotherapy according to the Non-Small Cell Lung Cancer, Version 1.2023, NCCN Clinical Practice Guidelines in Oncology [19], which recognize immunotherapy monotherapy as an appropriate option for patients unable to tolerate combination regimens.

**Fig. 1:**
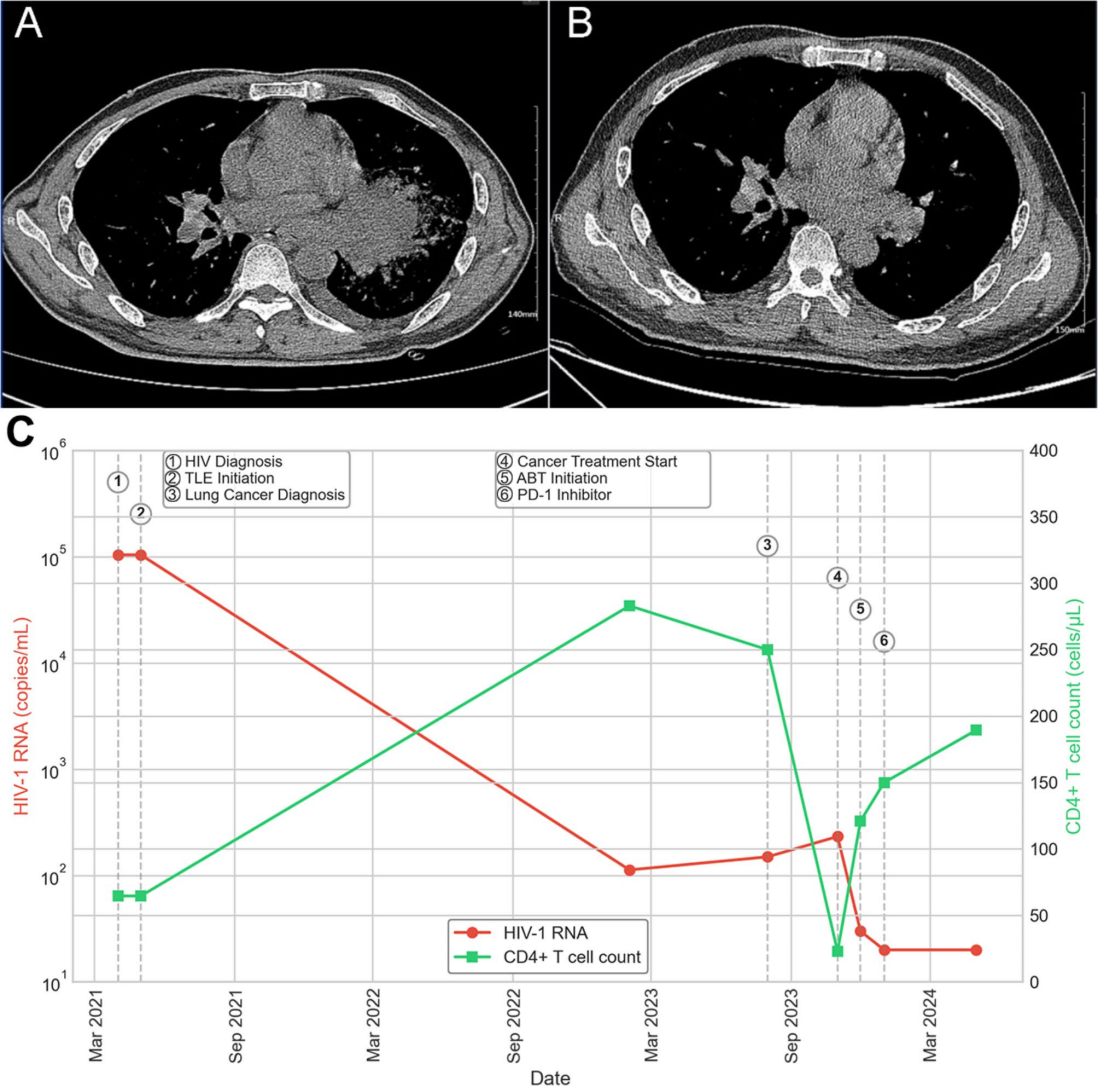
Serial chest CT imaging studies of Case 1 **A**: Contrast-enhanced chest CT (November 11, 2023) demonstrating a large left hilar mass (89 mm × 66 mm) with lobulation, spiculation, pleural indentation, and air bronchogram signs. Multiple nodular densities are present in both lungs with ground-glass opacities in the lower lobes. Enlarged bilateral hilar and mediastinal lymph nodes are visible (largest measuring 35 mm in long diameter). **B**: Follow-up chest CT (January 02, 2024) showing the evolution of the left hilar mass with associated changes in the surrounding lung parenchyma. **C**: Case 1 (Lung Cancer Patient). The graph demonstrates the temporal relationships between HIV diagnosis, initial antiretroviral therapy (TLE regimen), lung cancer diagnosis, cancer treatment initiation, ABT introduction, and subsequent modification to PD-1 inhibitor monotherapy. Note the rapid decline in viral load following ABT initiation despite severe immunosuppression related to cancer and its treatment. TLE: tenofovir disoproxil fumarate, lamivudine, and efavirenz; ABT: albuvirtide

By January 2024, the patient demonstrated marked clinical improvement. Follow-up imaging showed significant regression of inflammatory changes in the left hilar region and resolution of mediastinal lymphadenopathy (Fig. 1). The initiation of ABT corresponded with a rapid decline in HIV-1 RNA from 233 copies/mL to below the detection limit (< 20 copies/mL), despite the significant immunosuppression associated with advanced malignancy and cancer treatment. Concurrently, CD4 + T cell count (determined by flow cytometry) improved from a nadir of 22.82 cells/µL to 121 cells/µL, indicating successful viral suppression and partial immune reconstitution. As illustrated in Fig. 1C, this virological suppression was maintained throughout the extended follow-up period, with continued immunological recovery.

This virological suppression was sustained through May 2024, with continued immunological recovery evidenced by a progressive increase in CD4 + T cell count to 189.45 cells/µL at the most recent follow-up. Comprehensive safety monitoring was conducted throughout the treatment course. The previously documented chemotherapy-related adverse events (Grade 3 myelosuppression and Grade 2 peripheral neuropathy) showed gradual improvement during therapy, with ANC recovering from the lowest value of 0.8 × 10^9^/L to 1.6 × 10^9^/L by January 2024. Hepatic function parameters remained within normal range (ALT 32–45 U/L and AST 28–39 U/L), and renal function was stable with serum creatinine values of 0.8–0.9 mg/dL throughout the treatment period. No infusion-related reactions or injection site complications were observed with weekly ABT administration.

### Case 2: HIV-infected patient with breast cancer

A 61-year-old woman was initially diagnosed with HIV infection in November 2019, presenting with HIV-1 RNA of 7,670 copies/mL and a CD4 + T cell count of 191 cells/µL. She began antiretroviral therapy with tenofovir disoproxil fumarate, lamivudine, and efavirenz later that month. Despite consistent therapy, she maintained low-level viremia over the subsequent years. Genotypic resistance testing performed in October 2023 showed no major or minor resistance-associated mutations. Medication adherence evaluation revealed a suboptimal level of adherence, as indicated by a medication possession ratio of 0.76, primarily attributed to the patient’s reported concerns about potential side effects.

**Fig. 2:**
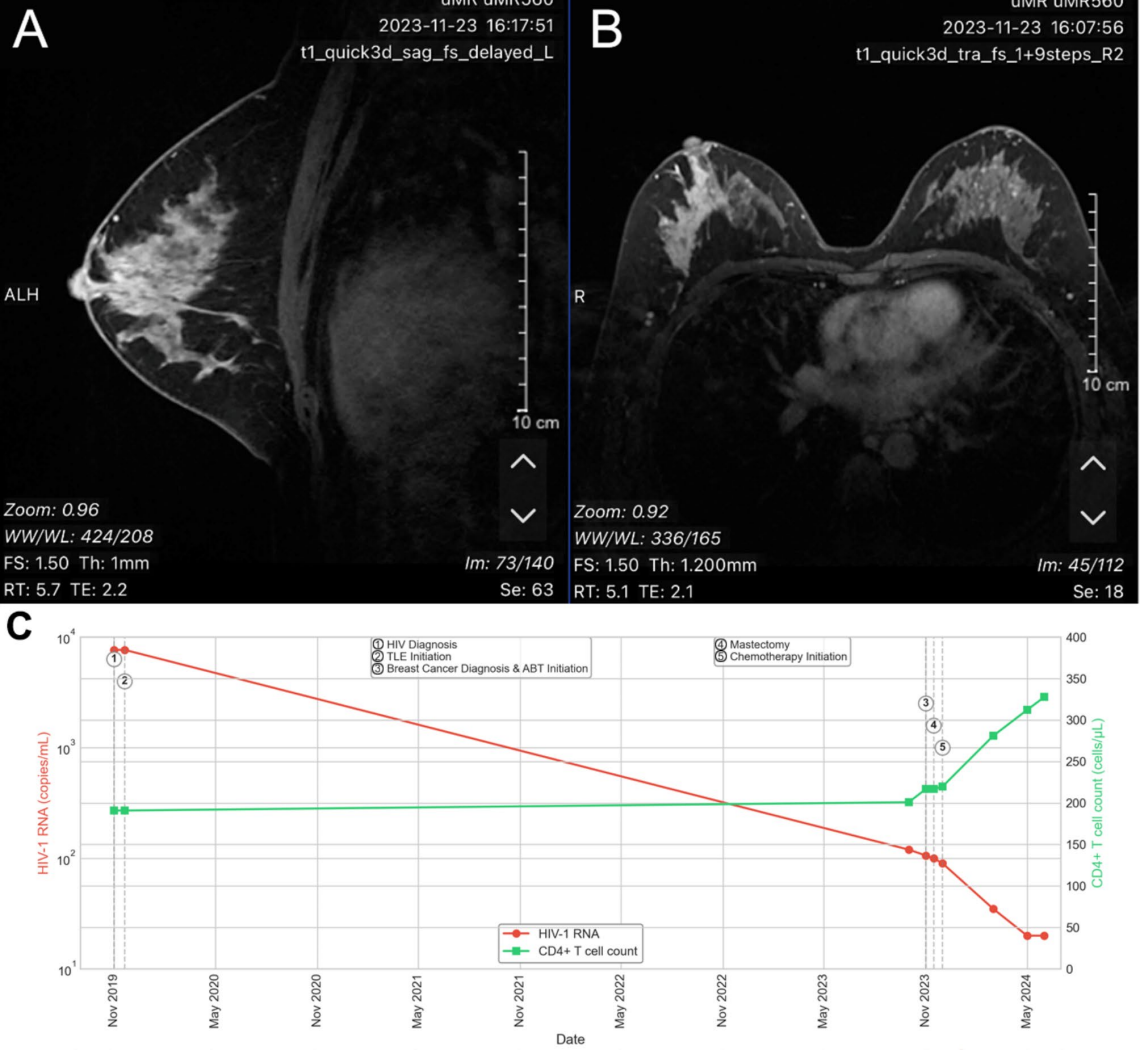
Breast imaging of Case 2 **A**: Contrast-enhanced breast MRI (November 23, 2023) demonstrating a large heterogeneous mass in the upper outer quadrant of the right breast (axial view). **B**: Contrast-enhanced breast MRI (November 23, 2023) showing the same lesion in sagittal view with characteristic malignant features including irregular margins and heterogeneous enhancement. **B**: Case 2 (Breast Cancer Patient). The timeline illustrates the relationships between HIV diagnosis, initial antiretroviral therapy, breast cancer diagnosis, ABT introduction, surgical intervention (mastectomy), and chemotherapy initiation. The gradual decline in viral load and steady improvement in CD4 + T cell count occurred despite concurrent cancer therapy. Vertical dashed lines indicate key clinical interventions. TLE: tenofovir disoproxil fumarate, lamivudine, and efavirenz; ABT: albuvirtide

In November 2023, she presented for evaluation of a right breast mass. Initial diagnostic workup included tumor marker analysis, revealing CEA 4.73 ng/mL, CA12-5 27.00 U/mL, CA19-9 4.42 U/mL, and CA15-3 13.39 U/mL. Contrast-enhanced breast MRI identified a 5.0 cm × 3.1 cm mass in the right breast’s upper outer quadrant, displaying characteristic features of malignancy (Fig. 2). A needle biopsy confirmed invasive carcinoma of no special type. Histopathological examination (November 2023) revealed high-grade invasive ductal carcinoma with extensive central necrosis, prominent nucleoli, and a high mitotic rate (15 mitoses per 10 high-power fields). The tumor cells demonstrated marked nuclear pleomorphism and infiltrative growth pattern, consistent with the diagnosis of invasive carcinoma of no special type, with immunohistochemistry demonstrating ER(-), PR(scattered cells weakly +), and HER-2(3+) status.

At this time, her HIV-1 RNA measured 106 copies/mL with a CD4 + T cell count of 217 cells/µL, indicating persistent low-level viremia. At the same time, She subsequently underwent right mastectomy with axillary lymph node dissection, with final pathology confirming stage IIB (pT2N1aM0) disease.

The patient received comprehensive therapy, including four cycles of epirubicin and cyclophosphamide followed by four cycles of albumin-bound paclitaxel with dual HER2-targeted therapy (trastuzumab and pertuzumab). Her antiretroviral therapy was optimized to include albuvirtide alongside tenofovir alafenamide, lamivudine, and efavirenz. The comprehensive management approach followed NCCN Guidelines for Breast Cancer (Version 4.2023) [20], incorporating appropriate surgical intervention, chemotherapy, and HER2-targeted therapy. Clinical monitoring showed improvement in both conditions: by March 2024, her HIV-1 RNA had decreased to 35 copies/mL with CD4 + T cell count increasing to 281 cells/µL, while tumor markers demonstrated significant improvement, with CEA decreasing to 1.26 ng/mL and CA15-3 to 22.40 U/mL by April 2024.

In November 2023, concurrent with her breast cancer diagnosis, the patient’s antiretroviral therapy was optimized to include albuvirtide (ABT) alongside tenofovir alafenamide, lamivudine, and efavirenz. Through continued follow-up to June 2024, further improvement in virological and immunological parameters was observed. By May 2024, the patient achieved complete viral suppression (HIV-1 RNA < 20 copies/mL) with CD4 + T cell count increasing to 312.24 cells/µL. The final recorded visit in June 2024 showed sustained viral suppression (HIV-1 RNA < 20 copies/mL) and a CD4 + T cell count of 327.83 cells/µL. The patient completed her planned chemotherapy regimen and continued HER2-targeted therapy per oncology recommendations. ABT administration was maintained on a weekly schedule, with excellent adherence reported throughout the treatment course. As shown in Fig. 2c, the introduction of ABT-containing therapy facilitated a progressive decline in HIV-1 RNA from 106 copies/mL to complete suppression by May 2024, accompanied by steady improvements in CD4 + T cell counts despite concurrent chemotherapy and targeted cancer therapy.

Comprehensive safety monitoring was conducted throughout the combined HIV and cancer treatment period. Hepatic transaminases showed mild elevations during chemotherapy (peak ALT 72 U/L, AST 65 U/L) but remained below grade 2 toxicity thresholds and normalized between cycles. Renal function remained stable (eGFR consistently > 80 mL/min/1.73 m²). Expected chemotherapy-related hematological effects (nadir ANC 0.9 × 10^9^/L, platelets 98 × 10^9^/L) were managed with standard supportive care without requiring dose reductions or delays. No significant drug-drug interactions were clinically apparent between ABT and the cancer treatment regimen.

## Discussion

The clinical management of HIV-associated low-level viremia represents a significant therapeutic challenge, particularly when complicated by NADC. Through these two cases, we provide initial observations regarding the potential utility of albuvirtide-containing antiretroviral therapy in this complex clinical scenario. While acknowledging the inherent limitations of case reports, these experiences offer valuable insights into several key clinical considerations.

Effective management of low-level viremia demands a comprehensive approach encompassing medication adherence optimization, resistance profiling, and careful monitoring of immune parameters. Contemporary guidelines advocate for antiretroviral regimen modification when viral loads consistently range between 50 and 1000 copies/mL, especially in the context of declining CD4 + T cell counts [21, 22]. In the two cases presented in this report, we observed that the integration of albuvirtide into existing antiretroviral regimens resulted in successful viral suppression, accompanied by CD4 + T cell count improvements despite concurrent immunosuppressive cancer therapy. Previous studies have shown that albuvirtide not only provides direct antiviral effects but may also offer immunomodulatory benefits for patients with suboptimal immune reconstitution [23], a characteristic particularly relevant for HIV patients undergoing cancer treatments.

The pathophysiology of LLV is multifactorial, involving ongoing viral replication in sanctuary sites, periodic virus release from latently infected cells, and adherence challenges [1, 24, 25]. Adding albuvirtide, a long-acting fusion inhibitor, offers distinct advantages in this context. By targeting the gp41 subunit of the HIV envelope glycoprotein and preventing the formation of the six-helix bundle structure necessary for viral fusion, ABT provides a complementary mechanism of action to traditional antiretrovirals [26, 27]. Its extended half-life of approximately 11–12 days enables once-weekly dosing, which significantly improves medication adherence, particularly during complex cancer treatment schedules [17, 28].

Clinical studies have demonstrated albuvirtide’s efficacy in treatment-experienced HIV patients, showing favorable virological suppression and CD4 + T cell recovery profiles [17, 23, 29, 30]. Recent evidence suggests ABT may provide immunomodulatory benefits beyond direct antiviral effects, potentially enhancing CD4 + T cell recovery in immunological non-responders [31]. This characteristic is particularly relevant for patients undergoing cancer treatments with immunosuppressive effects.

The complex interplay between HIV management and cancer treatment deserves special consideration [32]. Chronic immune activation associated with persistent viremia may compromise tumor surveillance mechanisms and affect cancer treatment outcomes [33]. The temporal relationship between ABT initiation and viral suppression in our cases suggests a positive impact, though confounding factors such as enhanced adherence monitoring during cancer care require consideration. Notable in our observations was the absence of significant drug-drug interactions between ABT and various cancer treatments (immunotherapy in Case 1; surgery, chemotherapy, and targeted therapy in Case 2), an important clinical advantage in this patient population.

It is important to consider alternative explanations for the observed virological and immunological changes [32]. Cancer therapy itself may affect HIV dynamics. For instance, in Case 1, PD-1 inhibitor therapy might have exerted complex effects—potentially reversing T cell exhaustion and enhancing immune-mediated viral control [34], yet possibly promoting latency reversal through CD4 + T cell activation [35]. Additionally, recent evidence suggests that expanded clones of HIV-infected CD4 + T cells infiltrating tumor tissues may contribute to low-level viremia in patients with malignancies [36]. This mechanism could be particularly relevant in our cases, as tumor-infiltrating lymphocytes harboring proviral DNA might experience altered activation states within the tumor microenvironment.

Several methodological limitations warrant consideration. The case report format precludes definitive conclusions regarding albuvirtide’s broader efficacy in this clinical context. The heterogeneous natural history of both HIV infection and cancer progression complicates the attribution of outcomes to specific therapeutic interventions. Additionally, while both patients achieved improved viral suppression, the relative contributions of albuvirtide versus other antiretroviral modifications remain unclear [37, 38].

These observations give rise to critical research questions for future investigation. Large-scale prospective studies are needed to evaluate albuvirtide-containing regimens in HIV-positive populations with LLV and concurrent malignancies, encompassing virologic and immunologic endpoints alongside cancer treatment responses and survival outcomes. Comprehensive pharmacokinetic analyses examining interactions between albuvirtide and diverse antineoplastic agents would provide essential guidance for treatment optimization. The relationship between viral suppression and cancer outcomes merits further exploration, particularly regarding whether effective cancer treatment modulates potential sources of viral production from tumor-infiltrating lymphocytes.

Implementation of albuvirtide-based therapy requires systematic consideration of practical clinical aspects, including integration of weekly administration into existing cancer treatment schedules and rigorous safety monitoring emphasizing overlapping toxicities between antiretroviral and cancer treatments. Prospective real-world studies evaluating these implementation strategies across diverse healthcare settings will help establish evidence-based best practices for this complex patient population.

## Data Availability

No datasets were generated or analysed during the current study.

## Declarations

### Data sharing

Study data are stored at Shishi Municipal Hospital. Proposals to access de-identified data can be submitted to zhangchuantiaoww@163.com after publication and, upon approval of the data request, a data use agreement will be set up before data transfer.

## Abbreviations

HIV: Human Immunodeficiency Virus
ABT: Albuvirtide ART Antiretroviral Therapy
CT: Computed Tomography
MRI: Magnetic Resonance Imaging
DWI: Diffusion-Weighted Imaging
ADC: Apparent Diffusion Coefficient
ER: Estrogen Receptor
PR: Progesterone Receptor
HER-2: Human Epidermal Growth Factor Receptor 2
CEA: Carcinoembryonic Antigen
CA: Cancer Antigen
